# Informationen zu Gesundheitsförderung und Prävention auf den Webseiten baden-württembergischer Gesundheitsämter – eine erste Annäherung

**DOI:** 10.1007/s00103-023-03818-w

**Published:** 2023-12-11

**Authors:** Jasmin Mangold, Daniela Hesmert, Achim Siegel, Anika J. Klein, David Häske, Sofie Wössner, Monika A. Rieger, Stefanie Joos, Cornelia Mahler

**Affiliations:** 1 Institut für Gesundheitswissenschaften, Abteilung Pflegewissenschaft, Universitätsklinikum Tübingen, Hoppe-Seyler-Straße 9, 72076 Tübingen, Deutschland; 2 Institut für Allgemeinmedizin & Interprofessionelle Versorgung, Universitätsklinikum Tübingen, Tübingen, Deutschland; 3 Institut für Arbeitsmedizin, Sozialmedizin und Versorgungsforschung, Universitätsklinikum Tübingen, Tübingen, Deutschland; 4 Zentrum für öffentliches Gesundheitswesen und Versorgungsforschung (ZÖGV), Universitätsklinikum Tübingen, Tübingen, Deutschland

**Keywords:** Öffentlicher Gesundheitsdienst, Gesundheitskommunikation, Internetauftritte, Inhaltsanalyse, Gesundheitsförderung und Prävention, Public health services, Health communication, Health promotion and prevention, Qualitative content analysis, Websites

## Abstract

**Hintergrund:**

Gesundheitsförderung und Prävention zählen zu den Kernaufgaben des Öffentlichen Gesundheitsdienstes. Von zentraler Bedeutung für ihre Wirksamkeit ist u. a. die Gesundheitskommunikation. Da sich das Internet zu einer wichtigen Quelle für Gesundheitsinformationen entwickelt hat und die Gesundheitsämter immer mehr im Interesse der Öffentlichkeit stehen, rücken auch ihre Webseiten mehr in den Fokus. Vor diesem Hintergrund wurde der Frage nachgegangen, wie Gesundheitsämter die Thematik der Gesundheitsförderung und Prävention nicht übertragbarer Erkrankungen (GuPnüE) über ihre Webseiten darstellen.

**Methoden:**

Die Webseiten der 38 baden-württembergischen Gesundheitsämter wurden von Juni bis Oktober 2022 mittels qualitativer Inhaltsanalyse untersucht. Die Darstellung der Thematik der GuPnüE über die Webseiten sowie präsentierte Maßnahmen der GuPnüE wurden erfasst. Es wurden die adressierte Zielgruppe, das Thema und die Interventionsform für jede Maßnahme erhoben.

**Ergebnisse:**

Auf allen Internetauftritten (*n* = 38) wurde die Thematik der GuPnüE aufgegriffen, die Darstellungsform war heterogen. Insgesamt wurden 243 GuPnüE-Maßnahmen über die 38 Internetauftritte identifiziert. Es zeigte sich ein breites Spektrum der in den dargestellten Maßnahmen bearbeiteten Themen, der adressierten Zielgruppen und genutzten Interventionsformen.

**Diskussion:**

Die Studie zeigt eine umfangreiche jedoch heterogene Darstellung der GuPnüE auf den Internetauftritten der Gesundheitsämter. Dabei bewegen diese sich im Spannungsfeld zwischen Anforderungen von Öffentlichkeitsarbeit und Gesundheitsinformation. Die Nutzung von Synergieeffekten durch die gemeinsame Bewerbung von überregional relevanten Materialien und Maßnahmen könnte Vorteile für die Gesundheitsämter mit sich bringen.

## Abstract (en)

**Background:**

Health promotion and prevention are core tasks of German public health services (Öffentlicher Gesundheitsdienst). Health communication is, among other things, central to their effectiveness. As the Internet has become an important source of health information and public health services are increasingly in the public eye, their websites are gaining more focus. We therefore investigated how public health services present topics on health promotion and prevention of non-communicable diseases (HPP-NCDs) on their websites.

**Methods:**

The websites of the 38 public health service departments in Baden-Wuerttemberg were examined using qualitative content analysis from June to October 2022. The presentation of the HPP-NCDs topic on the websites as well as the relevant measures were documented. For each measure/activity the addressed target group, the topic, and the type of intervention was collected.

**Results:**

The HPP-NCDs topic is addressed on all websites (*n* = 38); however, the presentation style is heterogeneous. A total of 243 HPP-NCDs measures/activities were identified across the 38 websites. There was a broad spectrum of topics, target groups, and types of intervention used in the measures/activities presented.

**Discussion:**

The study shows an extensive but heterogeneous presentation of HPP-NCDs on the websites of public health services. In doing so, they are caught between the requirements of public relations and health information. The use of synergy effects through the joint promotion of nationally relevant informational materials and measures could be beneficial for public health services.

## Hintergrund

Gesundheitsförderung und Prävention zählen neben der Gesundheitsplanung und -berichterstattung, den Gesundheitshilfen und dem Gesundheitsschutz zu den Kernaufgaben des Öffentlichen Gesundheitsdienstes (ÖGD; [1, 2]). Baden-Württemberg strebt in seinem Gesundheitsleitbild darüber hinaus eine Stärkung der Gesundheitsförderung und Prävention und eine Gleichstellung mit den Bereichen der medizinischen Versorgung und Pflege an [3]. Hiermit trägt das Bundesland der steigenden Zahl von Menschen mit nicht übertragbaren Erkrankungen und der hierdurch bedingten Relevanz dieser beiden Versorgungsbereiche Rechnung. Nicht übertragbare Krankheiten wie Herz-Kreislauf-Erkrankungen, chronische Atemwegserkrankungen, Krebs und Diabetes mellitus machen einen entscheidenden Teil der Krankheitslast der deutschen Bevölkerung aus [4]. Das Entstehen und der Verlauf dieser Erkrankungen können durch Maßnahmen der Gesundheitsförderung und Prävention positiv beeinflusst werden [5–8]. Hierzu gehören Maßnahmen der Verhältnis- und Verhaltensprävention, wie z. B. zur Steigerung der körperlichen Aktivität [8] und zur Veränderung der Ernährungsgewohnheiten in Kombination mit einer Steigerung der körperlichen Aktivität [5, 6].

In Baden-Württemberg sollen laut Gesetz über den Öffentlichen Gesundheitsdienst (ÖGDG; [1]) Gesundheitsförderung und Prävention zum Abbau von sozial bedingten Ungleichheiten im Bereich Gesundheitschancen beitragen (§ 7 ÖGDG Abs. 1). Der Kooperationsverbund Gesundheitliche Chancengleichheit hat mit den „Kriterien der guten Praxis für soziallagenbezogene Gesundheitsförderung“ [9] einen fachlichen Orientierungsrahmen für die Planung und Umsetzung gesundheitsförderlicher Maßnahmen entwickelt, die in diese Richtung wirken.

Gesundheitsförderung und Prävention stellen somit ein wichtiges Werkzeug dar, mit dem der ÖGD positiv auf die Bevölkerungsgesundheit einwirken kann. Allerdings mussten Aufgaben der Gesundheitsförderung und Prävention in den Gesundheitsämtern in Baden-Württemberg im Verlauf der Covid-19-Pandemie häufig zurückgestellt werden [10], während der Infektionsschutz in den Fokus der öffentlichen Aufmerksamkeit rückte.

Ein zentraler Bestandteil der Umsetzung von Gesundheitsförderung und Prävention ist die Gesundheitskommunikation [11]. Das Internet hat sich in den vergangenen Jahren zu einer wichtigen Quelle für gesundheitsbezogene Informationen entwickelt [13]. Die Studie von Horch [12] zeigt, dass das Internet bei der Suche nach Gesundheitsinformationen häufiger genutzt wird als alle anderen Medien. In einer deutschlandweiten Befragung aus dem Jahr 2019 gaben 31 % der Befragten an, bei der letzten Suche nach Gesundheitsinformationen das Internet genutzt zu haben [14]. Das Internet stand hier an zweiter Stelle direkt hinter der Beratung durch ärztliches und anderes medizinisches Personal und vor privaten Kontakten aus Familie und Freundeskreis als Informationsquelle. Das Vertrauen der Befragten in das Internet als Informationsquelle erwies sich jedoch als eher gering. So vertrauten nur 12,3 % der Befragten dem Internet stark oder sehr stark. Die Befragten vertrauen vor allem auf ärztliches Personal (40,8 % starkes; 37,5 % sehr starkes Vertrauen) sowie staatliche Gesundheitsbehörden (41,9 % starkes oder sehr starkes Vertrauen; [14]). Durch die Pandemie sind staatliche Gesundheitsbehörden, allen voran die Gesundheitsämter, und deren Webseiten verstärkt in den Fokus der Öffentlichkeit gerückt.

Vor diesem Hintergrund wurde der Frage nachgegangen, wie Gesundheitsämter die Thematik der Gesundheitsförderung und Prävention nicht übertragbarer Erkrankungen über ihre Webseiten darstellen.

## Methoden

### Setting.

Die Studie wurde als Teil des Kompetenznetzwerks Präventivmedizin Baden-Württemberg im Rahmen des Kompetenzbereichs Prävention und Öffentliches Gesundheitswesen und des Projekts „Prev4ÖGD“ vom Ministerium für Wissenschaft, Forschung und Kunst Baden-Württemberg gefördert (Förderkennzeichen: Az:04HV.MED(21)/11/1). Am Standort Tübingen wurden in diesem Rahmen unterschiedliche Aspekte der Umsetzung von Gesundheitsförderung und Prävention in den baden-württembergischen Gesundheitsämtern untersucht. Die vorliegende Studie war Bestandteil eines dieser Teilprojekte.

### Studiendesign.

Die Fragestellung wurde auf Grundlage einer Inhaltsanalyse der Webseiten der Internetauftritte^1^ der 38 baden-württembergischen Gesundheitsämter bearbeitet. Die Datenerhebung und -auswertung erfolgten mittels eines selbst entwickelten Leitfadens. Das Vorgehen orientierte sich dabei an der Forschungsfrage und den Merkmalen der qualitativen Inhaltsanalyse nach Schreier [15]. Die Berichterstattung der vorliegenden Studie orientiert sich insofern passend an den Empfehlungen der COREQ-Checkliste [16].

### Datengrundlage.

Als Datengrundlage dienten die Webseiten aller 38 baden-württembergischen Gesundheitsämter. Die Internetadressen wurden der vom Landesgesundheitsamt veröffentlichten „Liste der Gesundheitsämter Baden-Württemberg“ [17] entnommen. Wenn die angegebene URL nicht funktionierte, wurden in der Suchmaschine Google der „Ort“ und das Wort „Gesundheitsamt“ eingegeben und der Internetauftritt des Gesundheitsamtes aus der Trefferliste ausgewählt. Die Startseiten, inklusive der zugehörigen Unterseiten, die anhand ihrer Benennung Inhalte zu Gesundheitsförderung und Prävention nicht übertragbarer Erkrankungen (GuPnüE) anzeigten, wurden von einer der Autorinnen (DH, JM) mithilfe des Leitfadens auf ihre inhaltliche Relevanz geprüft. Die jeweilige Webseite wurde nur dann als relevant eingestuft und somit in die Studie einbezogen. Uneindeutige Fälle diskutierten die Autorinnen gemeinsam bis zu einer Konsensbildung. Die anhand dieses Verfahrens als relevant identifizierten Webseiten wurden lokal im Netzwerk des Universitätsklinikums im PDF-Format abgespeichert.

### Erhebungs- und Analyseinstrument.

Zur systematischen Erhebung und Analyse der Webseiten wurde ein Leitfaden entwickelt. Dieser diente bei der Erhebung zur Eingrenzung des Datenmaterials und bei der Analyse als Grundlage für das Kodieren des Datenmaterials. Die deduktiven Kategorien des Leitfadens wurden aus wissenschaftlichen Veröffentlichungen [18], grauer Literatur [9, 19, 20] sowie der Praxisdatenbank des Kooperationsverbundes Gesundheitliche Chancengleichheit [21] abgeleitet (Tab. 1). Nach einer ersten Durchsicht von 5 Internetauftritten und einem ersten Pretest an 3 Internetauftritten erfolgten jeweils eine Überarbeitung des Leitfadens und eine Ergänzung von induktiven Kategorien. Die Überarbeitung des Leitfadens erfolgte in einem iterativen Prozess in einem interprofessionellen und interdisziplinären Forschungsteam. Im Anschluss wurde der Leitfaden in einem Pretest von 2 unabhängigen Bewerterinnen (DH, JM) an 2 Internetauftritten getestet, der eine gute Übereinstimmung zwischen den Bewerterinnen zeigte.

**Table 1:** 

Kategorie	Arbeitsdefinition
Allgemeine Darstellung der Thematik	Form, in der die Thematik der GuPnüE auf der Startseite und den Unterseiten veranschaulicht wird
Handlungsfeld	Bereich des Handelns, in den die Maßnahme einzuordnen ist, angelehnt an die Bundesrahmenempfehlung nach § 20d Abs. 3 SGB V [32], abzuleiten anhand der gegebenen Informationen, eindeutige Zuordnung zu einer Unterkategorie
Zielgruppe	Personenkreis, auf dessen Gesundheit die Maßnahme einwirken soll. Sowohl direkt im Text benannte Gruppen wie auch aus den gegebenen Informationen sicher ableitbare Personengruppen werden erfasst. Zuordnung zu mehreren Unterkategorien möglich
Thema	Inhaltlicher Fokus der Maßnahme. Sowohl direkt im Text benannte Themen wie auch aus den gegebenen Informationen abgeleitete Themen werden erfasst. Zuordnung zu mehreren Unterkategorien möglich
Interventionsform	Im Rahmen der Maßnahme genutzte Form des Vorgehens. Sowohl direkt im Text benannte wie auch aus den gegebenen Informationen ableitbare Interventionsformen werden erfasst. Zuordnung zu mehreren Unterkategorien möglich

*GuPnüE* Gesundheitsförderung und Prävention nicht übertragbarer Erkrankungen

Mithilfe des Leitfadens und der darin enthaltenen Kategorien wurde die Darstellung des Themas GuPnüE und der dazugehörigen Maßnahmen erfasst. Unter einer „Maßnahme der GuPnüE“ wurde in dieser Studie eine über den Internetauftritt identifizierbare Tätigkeit des Gesundheitsamtes oder unter dessen Beteiligung im Bereich der GuPnüE verstanden. Angelehnt an das Verständnis des Kooperationsverbunds Gesundheitliche Chancengleichheit [9] waren hier auch Angebote, Projekte und Initiativen eingeschlossen. Das Angebot einer Beratung, die Teilnahme an einer Arbeitsgruppe, ein selbst erstellter Flyer wie auch ein komplexes Projekt wurden bei der Erhebung jeweils als eine einzelne Maßnahme gewertet und entsprechend kodiert. Ausgeschlossen wurden Tätigkeiten, zu denen es keinen erklärenden Informationstext gab, sowie Tätigkeiten, die eindeutig vor 2019 abgeschlossen waren. Das Jahr 2019 wurde hier als Grenze gewählt, um ein möglichst breites Bild der vor der Pandemie angebotenen Maßnahmen abbilden zu können und dennoch eine (gewisse) Aktualität der Maßnahmen zu gewährleisten. Flyer, auf deren Inhalte nicht zugegriffen werden konnte, waren ebenfalls ausgeschlossen. Die Teilnahme an überregionalen, von anderen Trägern initiierten Projekten (z. B. SunPass, Mädchen SUCHT Junge) wurde gesondert erfasst und nicht als Maßnahme gewertet.

### Datenerhebung und -analyse.

2 Autorinnen, DH (Allgemeinmedizin) und JM (Versorgungsforschung und Physiotherapie), führten die Datenerhebung und -analyse jeweils einzeln durch. Zu Beginn des Projekts hatten beide die Möglichkeit, über Hospitationen einen Einblick in die Arbeit in einem Gesundheitsamt zu erhalten. Die Datenerhebung fand im Zeitraum 01.06.2022 bis 24.10.2022 mit dem im Abschnitt „Erhebungsinstrument“ beschriebenen Vorgehen statt. Die Daten wurden anschließend inhaltsanalytisch ausgewertet. Es erfolgten eine Zuordnung relevanter Textabschnitte zu den bereits entwickelten Kategorien und Ausprägungen sowie eine Entwicklung von neuen Kategorien und Ausprägungen anhand des Datenmaterials. Freie Memos zu den einzelnen Internetauftritten und übergreifenden Themen ergänzten die Datenerhebung. Uneindeutige Textstellen diskutierten die beiden Autorinnen bis zur gemeinsamen Konsensbildung. Die Merkmale qualitativer Inhaltsanalyse nach Schreier [15] leiteten das Vorgehen.

Aufgrund des Erkenntnisinteresses erfolgte die quantitative Auswertung einiger Kategorien so, wie sie bspw. auch bei Bickelhaupt [22] und Gläser-Zikuda [23] durchgeführt wurde. Zur Unterstützung dieser quantitativen Auswertung wurden die entsprechenden Kategorien und ihre Ausprägungen in die Software Unipark (Tivian) überführt und später mit der Software SPSS (Version 28.0) deskriptiv ausgewertet. Bei der Ergebnisdarstellung in diesem Artikel wurde zur Wahrung der Anonymität auf die Bereitstellung von originalen Textabschnitten zur Untermauerung der Aussagen verzichtet.

## Ergebnisse

### Allgemeine Darstellung der Thematik GuPnüE.

Auf den Webseiten aller 38 untersuchten Internetauftritte wurde die Thematik der GuPnüE aufgegriffen, die Form der Darstellung war jedoch sehr heterogen. Auf den Startseiten aller Internetauftritte waren Oberbegriffe mit Verlinkungen zu finden, die auf Aktivitäten der GuPnüE schließen lassen. Besonders häufig wurden hier Oberbegriffe aus den Themengebieten „Kinder- und Jugendärztlicher Dienst“, „Zahnärztlicher Dienst“ und „Kommunale Gesundheitskonferenzen“ verwendet. Die Begriffe „Gesundheitsförderung“ und/oder „Prävention“ wurden auf 34 der 38 Internetauftritte wörtlich benannt. Sie konnten in 31 Fällen direkt auf der Startseite (0 Klicks) und in 3 Fällen auf nachgeordneten Webseiten (1 Klick) erreicht werden. Auf 18 der 38 Internetauftritte war eine spezifische Kontaktmöglichkeit (E-Mail-Adresse und/oder Telefonnummer) für den Bereich der Gesundheitsförderung und Prävention benannt.

Die Darstellungsform der Thematik GuPnüE über die Internetauftritte hinweg unterschied sich stark. So wurde auf einigen Internetauftritten in Stichworten aufgezählt, welche Tätigkeiten in diesem Bereich vom Gesundheitsamt durchgeführt werden. Auf anderen Internetauftritten wurden ausführliche Beschreibungen von aktuellen oder abgeschlossenen Projekten gegeben oder es wurden Gesundheitsinformationen zu spezifischen Erkrankungen, wie beispielsweise Diabetes mellitus, bereitgestellt. Die Webseiten der Gesundheitsämter sind meist in die Website des zugehörigen Landratsamtes, Landkreises oder der zugehörigen Stadt integriert.

### Dargestellte Maßnahmen der GuPnüE.

Auf den 38 Internetauftritten wurden insgesamt 243 Maßnahmen im Bereich GuPnüE identifiziert. Durchschnittlich konnten 6,39 (Mittelwert) Maßnahmen je Internetauftritt identifiziert werden (Median: 6, Spannweite: 0–16).

### Handlungsfelder der Maßnahmen.

Von den 243 Maßnahmen konnten 78 (32,1 %) dem Handlungsfeld „Gesund aufwachsen“ zugeordnet werden, 6 (2,5 %) dem Handlungsfeld „Gesundes Erwachsenenalter und Arbeiten“ und 14 (5,8 %) dem Handlungsfeld „Gesundes Altern“. Bei 145 (59,7 %) der identifizierten Maßnahmen war keine eindeutige Zuordnung zu einem der 3 Handlungsfelder möglich. In diesen Fällen waren die entsprechenden Maßnahmen altersgruppenübergreifend oder die bereitgestellten Informationen reichten nicht für eine eindeutige Zuordnung aus.

### Zielgruppen der Maßnahmen und Ansprache der Zielgruppe.

In den Informationstexten zu den einzelnen Maßnahmen wurde die Zielgruppe selten direkt angesprochen oder genannt. Häufig konnten jedoch anhand der gegebenen Informationen die von den Maßnahmen adressierten Zielgruppen geschlussfolgert werden. In einigen Fällen war dies jedoch nicht möglich oder die Maßnahme richtete sich an keine spezifische Zielgruppe, sondern an die allgemeine Bevölkerung.

Viele der präsentierten Maßnahmen nahmen unter anderem die Personengruppe der „Kinder“ (*n* = 65), der „Jugendlichen“ (*n* = 42) und der „werdenden und jungen Familien“ (*n* = 25) in den Blick (Tab. 2). Von den Zielgruppen mit besonderen Bedürfnissen wurden vor allem „chronisch Erkrankte“ (*n* = 21) sowie „Suchterkrankte“ (*n* = 16) durch die identifizierten Maßnahmen adressiert. Zahlreiche Maßnahmen zielten auf mehr als eine der genannten Zielgruppen ab.

**Table 2:** 

Zielgruppe^a^	Anzahl der zugeordneten Maßnahmen^b^
*Altersbezug*	
Kinder	65
Jugendliche	42
Ältere in der Kommune	12
Erwachsene	8
Auszubildende/Studierende	2
*Personen mit besonderen Bedürfnissen und Bedarfen*	
Chronisch Erkrankte	21
Suchterkrankte	16
Angehörige von Personen mit erhöhtem Unterstützungsbedarf	10
Personen mit kognitiven Einschränkungen	7
Personen mit Migrationshintergrund	5
Geflüchtete	5
Mobilitätseingeschränkte Personen	5
Personen mit Schwangerschaftskonflikt	5
Pflegebedürftige Personen	4
Personen ohne qualifizierten Schulabschluss	1
Personen mit niedrigem Einkommen/Rente	1
*Sonstige*	
Werdende und junge Familien	25
Mit Kindern/Jugendlichen Arbeitende (z. B. Lehrkraft, Fachkräfte)	3
Genderspezifisch (explizit Männer oder Frauen)	2
Sonstige Zielgruppen	26
Keine spezifische Zielgruppe angesprochen oder erkennbar	73
*Gesamtzahl Zuordnungen*^*b*^ ^*a*^	**338**

^a^ Folgende aus der Projektsuche des Kooperationsverbunds gesundheitliche Chancengleichheit übernommene Zielgruppen wurden bei keiner Maßnahme erkennbar als Zielgruppe anvisiert: Langzeitarbeitslose, Alleinerziehende, Personen aus strukturschwachen Wohnregionen, Personen mit prekären Arbeitsbedingungen, Personen mit niedrigem beruflichen Status, Personen in sozialer Isolation, Strafgefangene/Haftentlassene, Wohnungslose

^b^ Insgesamt 243 Maßnahmen, Mehrfachzuordnungen waren möglich

### Themen der GuPnüE.

**Figure 1:**
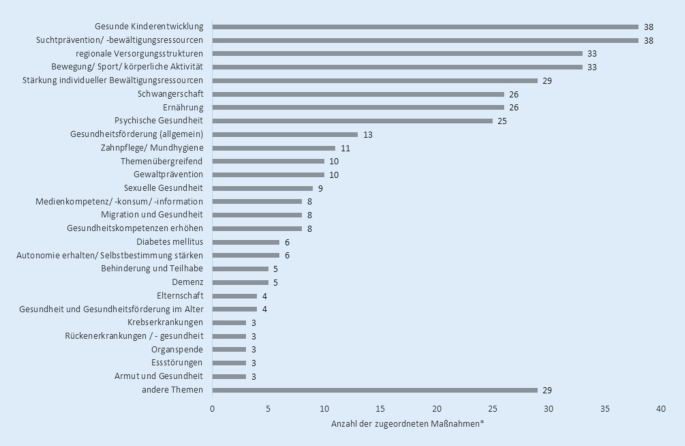

Die identifizierten Maßnahmen im Bereich GuPnüE betrafen besonders häufig die Themengebiete „Suchtprävention und Suchtbewältigungsressourcen“ (*n* = 38), „Gesunde Kinderentwicklung“ (*n* = 38), „Bewegung und körperliche Aktivität“ (*n* = 33) sowie „Regionale Versorgungsstrukturen“ (*n* = 33; Abb. 1). Ressourcenorientierte Themengebiete wie „Stärkung individueller Bewältigungsressourcen“ (*n* = 29), „Stärkung der Gesundheitskompetenz“ (*n* = 8) oder „Gesundheitsförderung (allgemein)“ (*n* = 13) wurden ebenfalls häufiger bearbeitet. Der Fokus lag aber seltener auf krankheitsorientierten Themen wie „Diabetes mellitus“ (*n* = 6), „Demenz“ (*n* = 5) und „Essstörungen“ (*n* = 3). Auch aktuelle Themen wie „Medienkompetenz und -konsum“ (*n* = 8) sowie Themen, die in Zusammenhang mit dem Ziel der gesundheitlichen Chancengleichheit stehen (Migration und Gesundheit (*n* = 8), Behinderung und Teilhabe (*n* = 5), Armut und Gesundheit (*n* = 3)), wurden im Rahmen der dargestellten Maßnahmen aufgegriffen. Zahlreiche Maßnahmen nahmen mehrere der genannten Themen in den Blick. Zu einigen Themen gab es von zahlreichen Ämtern Informationsmaterialien mit ähnlichen Inhalten, jedoch anderem Layout (bspw. zur Suchtprävention). Zu anderen Themen wurden von einigen der Gesundheitsämter nahezu identische Informationsmaterialien genutzt (bspw. Elternratgeber zur Einschulungsuntersuchung).

### Interventionsformen der Maßnahmen.

**Figure 2:**
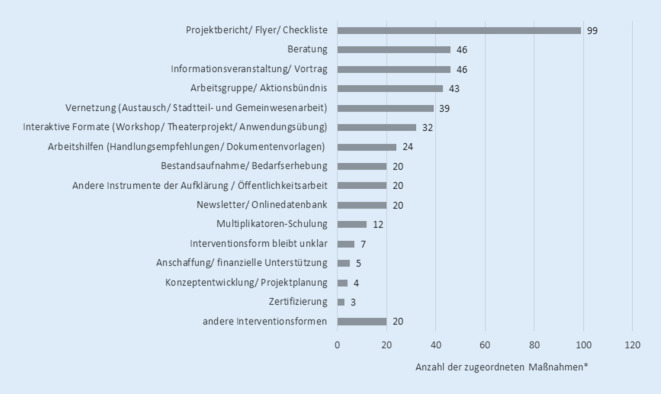

Das Spektrum der dargestellten Interventionsformen reichte hier von Einzelaktivitäten mit Aufklärungs- und Informationscharakter (z. B. Flyer, Newsletter oder einmalige Veranstaltungen) bis hin zu komplexen Interventionsprojekten, inklusive Evaluation (Abb. 2). Für viele Maßnahmen wurden personal- oder massenkommunikative Ansätze angewendet. Am häufigsten genutzt wurden hier u. a. Instrumente der Aufklärung und Öffentlichkeitsarbeit in schriftlicher Form (Projektberichte, Flyer, Checklisten, *n* = 99), gefolgt von einer mündlichen Information in Form von Vorträgen und Informationsveranstaltungen (*n* = 46) oder Beratungen (*n* = 46). In 20 Fällen beinhaltete die dargestellte Interventionsform eine Bedarfserhebung oder Bestandsaufnahme und in 12 Fällen wurde mit dem Instrument der Schulung von Multiplikatoren und Multiplikatorinnen gearbeitet.

## Diskussion

Diese Studie gibt einen ersten Einblick, wie die Thematik der GuPnüE über die Internetauftritte der baden-württembergischen Gesundheitsämter dargestellt wird. Sie zeigt, dass die Thematik auf allen Internetauftritten aufgegriffen wurde, jedoch in sehr heterogener Form. Es konnten insgesamt 243 GuPnüE-Maßnahmen über die 38 Internetauftritte hinweg identifiziert werden. Es zeigte sich ein breites Spektrum an Themen, Zielgruppen und Interventionsformen.

### Darstellung der Thematik und Spektrum der präsentierten Maßnahmen

Die beschriebene Heterogenität zeigte sich vor allem in der Darstellungsform, diese reichte von einer stichpunktartigen Aufzählung von Aufgaben bis zu ausführlichen Projektbeschreibungen und der Bereitstellung von Gesundheitsinformationen. Diese starke Heterogenität könnte durch den unterschiedlichen Zweck, den die Internetauftritte der einzelnen Gesundheitsämter erfüllen, bedingt sein. So können Internetauftritte von Gesundheitsämtern sowohl für deren Öffentlichkeitsarbeit als auch für die Gesundheitskommunikation und -information genutzt werden.

Es zeigte sich, dass zahlreiche Maßnahmen die Zielgruppen „Kinder“ und „Jugendliche“ adressieren. Dies könnte durch die expliziten gesetzlichen Vorgaben in diesem Bereich bedingt sein. Im ÖGDG [1] wird die Beratung von Kindern, Schülerinnen und Schülern, sorgeberechtigten Personen sowie Kindertageseinrichtungen und Schulen zu erforderlichen Maßnahmen der Gesundheitsförderung und Prävention durch Gesundheitsämter explizit aufgeführt (§ 8 ÖGDG Abs. 1). Weiter gab es Hinweise auf eine Fokussierung auf die Zielgruppe „werdende und junge Familien“. In der Studie von Klein et all. [24] zeigte sich, dass die Themensetzung in der Gesundheitsförderung und Prävention in Gesundheitsämtern durch politische Akteurinnen und Akteure beeinflusst sein kann. Junge Familien sind ein wichtiger Zukunftsfaktor für Kommunen – eine politische Einflussnahme auf die dargestellten Maßnahmen im Bereich GuPnüE wäre daher denkbar.

Da sich soziale Unterschiede schon in der gesundheitlichen Entwicklung von Kindern und Jugendlichen zeigen und die Weichen für das Gesundheitsverhalten bereits früh gestellt werden [25], sollten Maßnahmen der Gesundheitsförderung früh ansetzen. Dies könnte ebenfalls eine Fokussierung auf die oben genannten Gruppen erklären. Jüngere Personen nutzen zudem das Internet u. a. zur Information über sensible Themen wie Drogen, psychische Erkrankungen und sexuelle Gesundheit [26–28]. Dass sie von vertrauenswürdigen Informationen zu diesen Themen besonders profitieren können, ist wahrscheinlich ein weiterer Grund für das häufigere Angebot solcher Informationen. Auf den Webseiten der Gesundheitsämter wurden bereits zahlreiche Maßnahmen dargestellt, welche die Themen Sucht- und Suchtbewältigung sowie psychische Gesundheit thematisieren. Auch die Themen Gewaltprävention und sexuelle Gesundheit wurden in einigen der dargestellten Maßnahmen bearbeitet.

Weiter zeigte sich, dass krankheitsorientierte Themen seltener im Rahmen der dargestellten Maßnahmen bearbeitet wurden als ressourcenorientierte Themen. Dies könnte einerseits durch die in der Studie gewählte Methodik verursacht sein, könnte nach Meinung der Autorinnen und Autoren aber auch ein Hinweis auf einen Paradigmenwechsel in den Gesundheitsämtern sein: weg von einer Defizitorientierung, hin zu einer Ressourcenorientierung.

Bei den bzgl. der Maßnahmen angewendeten Interventionsformen zeigt sich ein Trend hin zu massenkommunikativen Ansätzen wie Flyern, Berichten und Checklisten, möglicherweise weil diese ressourcenschonend in der Erstellung sind und gut im Internet dargestellt werden können. Verhältnispräventive Maßnahmen kommen aktuell auf den Webseiten kaum zum Ausdruck. Sie lassen sich vermutlich schwerer öffentlichkeitswirksam präsentieren als Maßnahmen der Verhaltensprävention, da sie häufig aus Maßnahmenbündeln bestehen, die unterschiedliche Zielgruppen und Stakeholder adressieren müssen, um wirksam zu werden.

Die vom Kooperationsverbund Gesundheitliche Chancengleichheit aufgestellten Kriterien der guten Praxis der soziallagenbezogenen Gesundheitsförderung fordern unter anderem die Anwendung des Multiplikatoren-Konzepts und eine nachhaltige Gesundheitsförderung. Zur Sicherstellung der Nachhaltigkeit werden eine bedarfsorientierte Maßnahmenkonzeption und -planung, ein Wirksamkeitsnachweis sowie die Verstetigung bzw. im besten Fall die kontinuierliche Weiterentwicklung der verstetigten Maßnahme angestrebt [9]. Über die Webseiten konnten 12 Maßnahmen identifiziert werden, in deren Rahmen Multiplikatoren-Schulungen durchgeführt werden, und in 20 Fällen war eine Bedarfserhebung sichtbar. Es zeigte sich somit, dass im Rahmen einiger Maßnahmen bereits Kriterien der soziallagenbezogenen Gesundheitsförderung umgesetzt und präsentiert wurden. Potenzial für eine verstärkte Präsentation der Kriterien wurde jedoch ebenfalls sichtbar.

### Nutzung des Internets zur Gesundheitskommunikation

Das Internet stellt ein niederschwelliges und schnelles Medium der Gesundheitskommunikation mit enormer Reichweite dar [12], welches von vielen Menschen in Deutschland bei der Suche nach Gesundheitsinformationen genutzt wird [13]. Nachteile des Internets liegen in der mangelnden Übersichtlichkeit und den fehlenden Qualitätsstandards [12]. Hier liegt vermutlich ein Grund für das mangelnde Vertrauen der deutschen Bevölkerung in das Internet als Informationsquelle. Durch das Vertrauen, das Gesundheitsbehörden genießen [14], könnte den Internetauftritten von Gesundheitsämtern nach Meinung der Autorinnen und Autoren hier jedoch eine Sonderstellung zukommen. Es könnten diese eine Möglichkeit darstellen, mit der Bevölkerung zielgerichtet zu Themen der Gesundheitsförderung und Prävention zu kommunizieren. So können sowohl Gesundheitsinformationen bereitgestellt, konkrete Maßnahmen der Gesundheitsförderung und Prävention beworben, wie auch Möglichkeiten der Partizipation geschaffen werden. Die Studie zeigte, dass diese Möglichkeiten von einigen Gesundheitsämtern im Bereich der GuPnüE bereits genutzt werden, für andere Ämter ein Ausbau des Internetauftritts in diesem Bereich sicher noch möglich ist. Ein erster Schritt könnte hier eine bewusste Entscheidung sein, den Internetauftritt auch tatsächlich zum Zweck der Gesundheitsinformation zu nutzen. Mit dieser Entscheidung wird ein Spannungsfeld zwischen den verschiedenen Anforderungen eröffnet, die an Öffentlichkeitsarbeit und an die Publikation von Gesundheitsinformationen gestellt werden. Je nachdem, welchem Zweck der entsprechende Inhalt dienen soll, wird sich dessen Ausgestaltung an den entsprechenden Empfehlungen für eine zielgerichtete Gesundheitsinformation bzw. effektive Öffentlichkeitsarbeit orientieren müssen.

Bei der Bereitstellung von Gesundheitsinformationen über das Internet sollte beachtet werden, dass Menschen mit niedrigerem sozioökonomischen Status seltener nach Gesundheitsinformationen suchen [26, 29] und daher die Gefahr einer Verstärkung der gesundheitlichen Ungleichheit besteht [30]. Einige Studien verweisen in diesem Zusammenhang auf eine ausgleichende Wirkung des Internets, wohingegen andere auf eine Verstärkung von Wissensunterschieden hinweisen [30]. Eine verstärkte Orientierung an den Kriterien der soziallagenbezogenen Gesundheitsförderung könnte helfen, trotz dieser aktuellen Unsicherheit dem Auftrag des Abbaus von sozial bedingten Ungleichheiten von Gesundheitschancen gerecht zu werden.

Da das Internet von jüngeren Personen u. a. zur Information über sensible Themen wie sexuelle und psychische Gesundheit, Suchtbewältigung und Gewaltprävention genutzt wird [27, 28], bietet es sich an, die Webseiten der Gesundheitsämter zur Bereitstellung vertrauenswürdiger Informationen und Angebote zu nutzen. Die Ergebnisse zeigen, dass solche sensiblen Themen in einigen der von den Gesundheitsämtern dargestellten Maßnahmen bereits bearbeitet und präsentiert wurden und ein Anfang hier bereits gemacht ist. Jugendliche bewegen sich vermehrt in sozialen Medien, weshalb die Gesundheitskommunikation durch Gesundheitsämter über diese Medien ebenfalls diskutiert wird. Hier kann zukünftige Forschung helfen, den tatsächlichen Nutzen sozialer Medien für eine zielgerichtete Gesundheitskommunikation zu klären, um diese Entscheidung evidenzbasiert treffen zu können. Aktuell ist nicht bekannt, welche Faktoren in den Gesundheitsämtern Einfluss auf die Darstellung von GuPnüE auf den Internetauftritten nehmen. Zudem ist unklar, wie die Webseiten dort verwaltet werden und wer als Zielgruppe gesehen wird. Die Studie hat gezeigt, dass die Webseiten der Gesundheitsämter meist in die Website des zugehörenden Landkreises, der Stadt oder des zugehörenden Landratsamtes integriert sind. Unklar bleibt dabei, ob dies einen Einfluss auf die Anzahl der zur Verfügung stehenden Webseiten oder deren Inhalte hat. Weitere Forschung mit einem Fokus auf diese Aspekte könnte helfen die dargestellten Ergebnisse tiefergehend zu interpretieren. Die dargestellte Heterogenität der Internetauftritte spiegelt sich auch in den Strukturen der Gesundheitsämter, wie in den anderen Teilstudien des Projekts Prev4ÖGD festgestellt werden konnte [10, 31].

### Limitationen

Die Durchführung der Studie war mit konzeptionellen und methodischen Herausforderungen verbunden. Trotz der vorgenommenen Eingrenzung auf GuPnüE gestaltete sich die inhaltliche Abgrenzung des Bereichs der GuPnüE angesichts heterogener Definitionen und Abgrenzungen der Begriffe Gesundheitsförderung und Prävention schwierig [32, 33]. Zudem war die Anwendung der aus der Theorie stammenden Definitionen auf die in der Praxis durchgeführten Maßnahmen eine Herausforderung. Diesen Herausforderungen wurde begegnet, indem im Erhebungsinstrument gegenstandskonkretisierende Vorgaben ergänzt wurden. Durch das gewählte Vorgehen ist eine Verzerrung der Ergebnisse nicht auszuschließen. Der maßnahmenbezogene Zugang eröffnete aufgrund der unterschiedlichen Komplexität der Maßnahmen die Herausforderung der Zusammenfassung und Gewichtung, die nicht abschließend aufgelöst werden konnte: So wurde eine Interventionsstudie im Themenbereich der Ernährung gleich gewichtet wie ein Flyer im Themenbereich der Bewegung, obwohl der personelle Aufwand und die anzunehmende Wirkung sich unterscheiden. Quantitativ vergleichende Aussagen können daher nur eingeschränkt vorgenommen werden. Qualitative Aussagen zum Spektrum der Maßnahmen sowie vorsichtige Trendaussagen sind dennoch möglich. Die Ergebnisse stellen eine Momentaufnahme aus Baden-Württemberg dar. Es kann vermutet werden, dass andere gesetzliche Grundlagen sowie Verwaltungsstrukturen ein abweichendes Bild in den anderen Bundesländern zeigen. Eine Übertragung auf andere Bundesländer oder ganz Deutschland ist nicht möglich.

Zudem sollte beachtet werden, dass bisher wenig zu den Erwartungen der Nutzenden an die Internetauftritte und deren tatsächliche Nutzung durch verschiedene sozioökonomische Gruppen bekannt ist. Insbesondere zur Auffindbarkeit von relevanten Informationen und deren Verständlichkeit bräuchte es eine vertiefende (z. B. kommunikationswissenschaftliche) Betrachtung, die in dieser Studie nicht im Fokus stand.

## Fazit

Die vorliegende Studie zeigt, dass GuPnüE auf den Internetauftritten der Gesundheitsämter in Baden-Württemberg umfangreich dargestellt wird, wobei eine starke Heterogenität in der Darstellung und inhaltlichen Schwerpunktsetzung der Thematik auffällt. Die Internetauftritte von Gesundheitsämtern bewegen sich in dem Spannungsfeld der Anforderungen einer wirksamen Öffentlichkeitsarbeit und einer zielgerichteten Gesundheitsinformation. Der Klärung der Frage, welchem dieser beiden Zwecke der konkrete Inhalt vorrangig dienen soll, kommt daher bei der Gestaltung des Internetauftritts eine besondere Bedeutung zu. Hier wird entschieden, welchen Anforderungen die Darstellung der Inhalte entsprechen muss. Bei der Bereitstellung von Gesundheitsinformationen mit überregionaler Relevanz und der Bewerbung von überregionalen Maßnahmen könnten über eine vermehrte Nutzung von gemeinsamen (evidenzbasierten) Informationstexten Vorteile für die einzelnen Gesundheitsämter generiert werden.

## Footnotes

1 Zur begrifflichen Klärung: Ein „Internetauftritt“ besteht aus allen „Webseiten“, die das Gesundheitsamt befüllt. Als „Website“ wird eine abgeschlossene Domäne verstanden.
